# Urinary polycyclic aromatic hydrocarbon metabolites are positively related to serum testosterone levels of males and serum estradiol levels of females among U.S. adults

**DOI:** 10.3389/fendo.2022.1037098

**Published:** 2022-12-07

**Authors:** Qiming Yuan, Kun Jin, Xianghong Zhou, Zhimei Qiu, Jiakun Li, Di Jin, Zilong Zhang, Chichen Zhang, Lu Yang, Yu Zhan, Shi Qiu, Qiang Wei

**Affiliations:** ^1^ Department of Urology, Institute of Urology, West China Hospital of Sichuan University, Chengdu, China; ^2^ Department of Environmental Science and Engineering, Sichuan University, Chengdu, Sichuan, China

**Keywords:** polycyclic aromatic hydrocarbon, testosterone, estradiol, reproductive health, national health and nutrition examination survey

## Abstract

**Background:**

It has been reported for several years that polycyclic aromatic hydrocarbons (PAHs) could disturb human endocrine function. However, there is still a short of consistent conclusion about the relationship between PAH exposure and levels of sexual hormones. The aim of our study is to explore whether exposure to PAHs and how PAHs affect the levels of serum testosterone (T) and estradiol (E2) in adults, hoping to fulfill the knowledge gap.

**Materials and methods:**

This study included adults aged 20 and above who participated in the National Health and Nutrition Examination Survey (NHANES) from 2011 to 2016. We included 10 PAH metabolites in this study. The levels of urinary PAH metabolites were log-transformed and divided into quartiles. The associations between PAH metabolites and both serum T levels of males and E2 levels of females were investigated using multivariate regression models. We furtherly calculated PAHs scores by sum of ranks across 10 PAHs metabolites, which represented the exposure levels of PAHs mixtures, and the association between PAHs scores and serum T and E2 levels were analyzed.

**Results:**

A total of 4,654 subjects were included in this study, including 2,460 males and 2,194 females. After adjusting for confounders, 2-hydroxynapthalene and 3-hydroxyfluorene were positively associated with serum T levels of males (p-value for trend=0.047, and p-value for trend=0.006, respectively), while 1-hydroxyphenanthrene was positively associated with serum E2 levels of females (p-value for trend=0.013). In the adjusted models, no significant association was found between PAHs scores and either T levels of males or E2 levels of females (p-value for trend=0.615, and p-value for trend=0.241, respectively).

**Conclusions:**

This study showed urinary 2-hydroxynapthalene and 3-hydroxyfluorene were associated with increased T levels of males, and urinary 1-hydroxyphenanthrene was associated with increased E2 levels of females. The observed association indicated disrupting effects of PAH exposure on reproductive health.

## Introduction

Polycyclic aromatic hydrocarbons (PAHs) are a family of environment toxin which are ubiquitous in urban atmospheres. PAHs are produced mainly by incomplete combustion of organic compounds, including diesel, gasoline, coal, oil, wood, et al. (1), and people can expose to PAHs through smoking, vehicle exhaust, industrial production, and food (2). To evaluate the exposure level of people to PAHs, previous studies mainly applied levels of urinary PAH metabolites (3–6). Studies have reported environment toxins could disturb the levels of reproductive hormone and increase the risk of relative diseases such as breast cancer (7–10). As an important environment toxin, the relationship between PAHs and people’s reproductive system is under intense investigation.

Testosterone (T) as the major male sex hormone can promote the development of male reproductive tissue and spermatogenesis. The secretion of T is regulated by luteinizing hormone (LH), which is secreted at anterior pituitary responded to gonadotropin-releasing hormone (GnRH). A previous study found PAHs could disturb the balance of hypothalamic-pituitary-gonadal axis, and in this study a positive association between secretion of GnRH and exposure to low-dosage PAHs was detected, which led to an increase of serum T level (11). However, another study reported that PAHs may disturb the function of Leydig cells, which are a group of cells in testis secreting T, resulting in a decrease of T in serum (12).

Estradiol (E2), as the most abundant and most active estrogen, is secreted by granulosa cells of follicles in the ovary. E2 plays an important role in regulating women’s somatic and psychological health. For example, women will show increased susceptibility to nausea and motion sickness during the early (low serum E2 level) versus late follicular (high serum E2 level) phase (13, 14). One previous study found exposure to PAHs could enhance the production of reactive quinone species of endogenous estrogen, and there was a positive correlation between PAHs exposure and activation of estrogen, including E2 (15). However, another study found PAHs could decrease E2 secretion by disturbing the function of granulosa cells *via* ESR1 and GPER1 receptors (16).

Although the association between PAH exposure and people’s reproductive health has aroused more and more attention, there is still a short of consistent conclusion about the relationship between PAH exposure and levels of sexual hormones. This may attribute to geographical differences, racial differences, selected PAHs biomarkers and exposure levels. In addition, as present studies mainly performed on animals or *in vitro* (17–21), studies investigating the association directly among human are still lacking. Thus, the aim of our study is to explore whether exposure to PAHs and which PAH could affect the levels of serum T and E2 in adults, hoping to fulfill the knowledge gap.

## Materials and methods

### Study subjects

**Figure 1:**
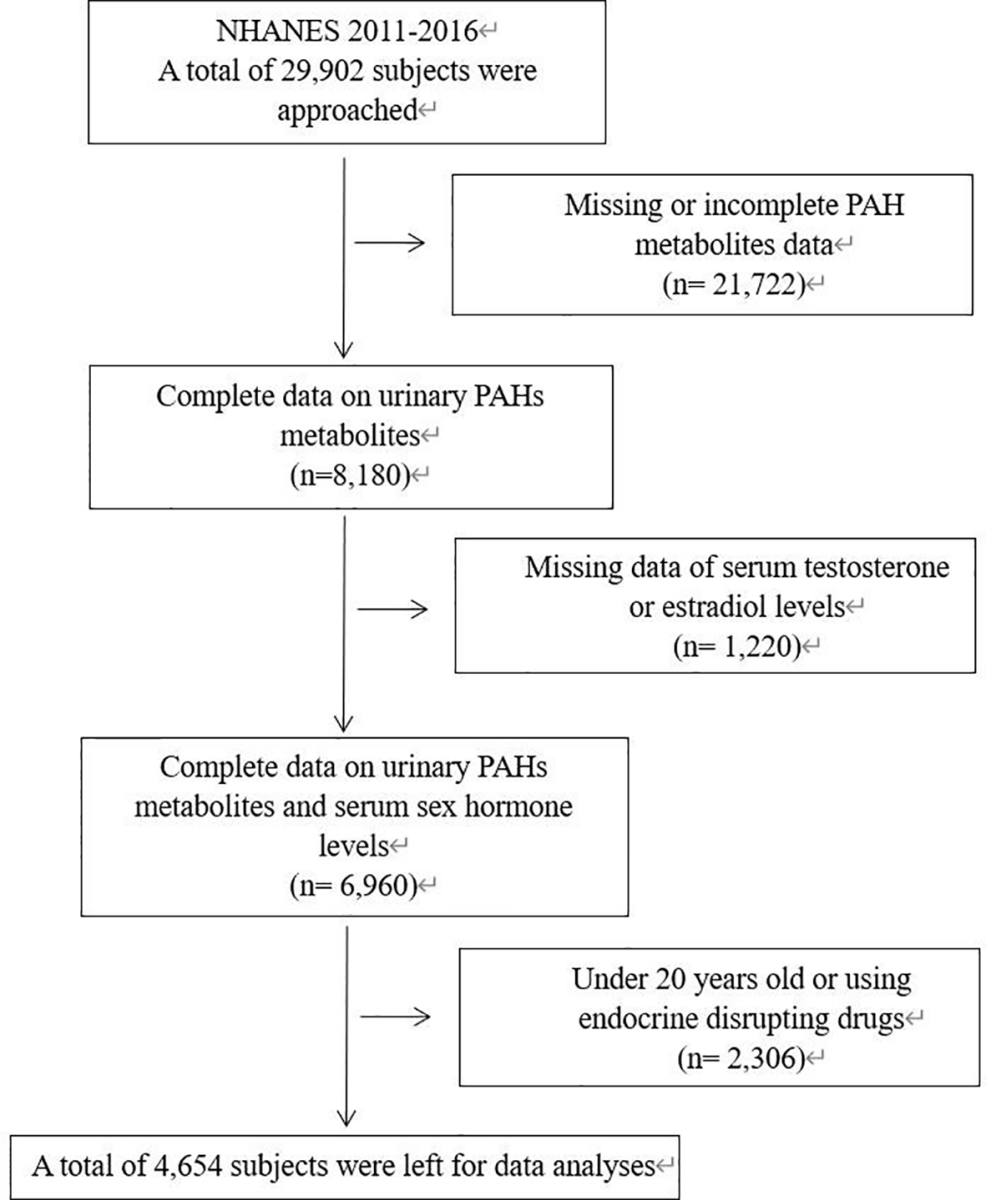
Flowchart describing the selection of patients. NHANES, National Health and Nutrition Examination Survey; PAH, Polycyclic aromatic hydrocarbons.

The study subjects were selected from participants of the National Health and Nutrition Examination Survey (NHANES) from 2011 to 2016. The NHANES is a cross-sectional survey of non-institutionalized citizens conducted by NCHS of the Centers for Disease Control and Prevention. The data of participants such as race, income status, dietary condition and medical history was collected through household interviews. Meanwhile, participants would undergo physical examination, and blood and urine would be collected at specific examination centers. All participants were given informed consents, and the NHANES protocol was approved by the National Center for Health Statistics Research Ethics Review Board. We included subjects with complete urinary PAHs metabolites levels and complete serum T and E2 levels. As there was a significant difference in sexual hormone levels between minors and adults, we only included adults aged 20 and above. Subjects using endocrine disrupting drugs were furtherly excluded. A total of 4,654 subjects were included in our study, including 2,460 males and 2,194 females. Details of inclusion and exclusion criteria were shown in **Figure 1**.

### Urinary PAH metabolites measurement

The urine samples were collected at mobile examination center by trained professionals. After the collection, urine samples were stored at a temperature of -20°C or lower and transported to National Center for Environmental Health for analysis. The glucuronidated or sulfated OH-PAH metabolites in urine experienced enzymatic hydrolysis, extraction, and derivatization. Then the values of metabolites were measured using isotope dilution capillary gas chromatography tandem mass spectrometry (GC-MS/MS). The detailed information about detection and measurement of urine PAH metabolites was provided in the NHANES laboratory method (http://www.cdc.gov/nchs/data/nhanes/nhanes_11_12/PAH_G_met.pdf). We included 10 PAH metabolites in this study: 1-hydroxynapthalene (1-naphthol), 2-hydroxynapthalene (2-naphthol), 3-hydroxyfluorene, 2-hydroxyfluorene, 3-hydroxyphenanthrene, 1-hydroxyphenanthrene, 2-hydroxyphenanthrene, 1-hydroxypyrene, 9-hydroxyfluorene, and 4-hydroxyphenanthrene. These 10 PAHs metabolites are major monohydroxylated metabolites of four important PAHs, including naphthalene, fluorene, phenanthrene, and pyrene. In the NHANES database from 2011 to 2016, only these 10 metabolites were available.

### Serum T and E2 measurements

The collected serum samples were stored at a temperature of -20°C or lower until they were transported to the Division of Environmental Health Laboratory Sciences for analysis. Before the quantitative analysis, serum T and E2 were dissociated from binding proteins, extracted from sample matrix, and potentially interfering compounds were also removed. Then, serum T levels and E2 levels were measured using isotope dilution liquid chromatography tandem mass spectrometry (ID-LC-MS/MS) by the CDC (https://wwwn.cdc.gov/Nchs/Nhanes/2013-2014/TST_H.htm).

### Statistical analysis

To perform the analyses, we used EmpowerStats (http://www.empowerstats.com, X&Y Solutions, Inc., Boston, MA) and statistical software packages R (http://www.R-project.org, The R Foundation). A p value of <0.05 was considered statistically significant. The continuous variables were presented as mean ± standard deviation, and the categorical variables were presented as the frequency and its proportion. When the values of PAH metabolites were below the limit of detection, the values were reserved as the detection limit divided by the square root of two. The following categorical variables were included: race (Mexican American, Other Hispanic, Non-Hispanic White, Non-Hispanic Black, Other Race), ratio of family income to poverty (<1.3, 1.3-3.5, >3.5), education level (Less than high school, High school or General educational development, Above high school), marital (Married or living with partner, Living alone), BMI (<=25, >25 and <=30, >30), comorbidity index (0, 1, 2–5), smoking (never, former, current), alcohol intake (none, moderate, heavy). The levels of 10 urinary PAH metabolites were log-transformed to acquire a normal distribution, and the geometric mean levels of creatinine-corrected urinary PAH metabolites were divided into quartiles. The first quartile (lowest level) was set as the reference group, and the β-coefficient and 95% confidence interval (CI) were analyzed using linear regression model to determine whether there was a dose-response relationship between PAH exposure and serum T levels of males and E2 levels of females. P-value for trends were calculated to evaluate the statistical significance of trends. Extended models were performed to adjust covariates, including age, race, BMI, time of venipuncture, ratio of family income to poverty, education level, marital, comorbidity index, smoking, and alcohol intake. As the levels of T and E2 vary physiologically in relation to age, we furtherly performed age-stratified analysis. Subjects were divided into three groups: <40, >=40 and <60, >=60 groups. Associations between PAHs metabolites and sex hormones were tested in the three age groups separately. The association between PAHs metabolites and sex hormone binding globulin (SHBG) levels of males was also analyzed. Additionally, to investigate the association between exposure to PAHs mixtures and serum sexual hormone levels, we sorted the 10 urinary PAH metabolites from lowest to highest to create ranks separately, and PAHs scores were established by sum of ranks across 10 urinary PAH metabolites for each participant (22). High value of PAHs score signified heavy burden of exposure to PAHs mixtures. Association between PAHs scores and serum T and E2 levels were further investigated. Age-stratified analysis was also performed between PAHs scores and sex hormones.

## Results

### Characteristics of subjects

Demographic characteristics, serum T and E2 levels of 4,654 subjects from NHANES 2011-2016 were shown in **Table 1**. The mean age of male subjects was 48.88 years old, while females were 46.29. The BMI of 33.58% male subjects were over 30 kg/m^2^, and the BMI of 41.75% female subjects were over 30 kg/m^2^. For males, smokers, including former and current smokers, were up to 52.56% of subjects, while the percentage of females was 32.68%. The mean T and SHBG levels of male subjects were 409.29 ng/dl and 44.41 nmol/l, while the mean E2 level of female subjects was 82.91 pg/ml.

**Table 1:** Demographic and laboratory characteristics of 4,654 subjects.

Gender	Male (n = 2460)	Female (n = 2194)
Age, year, mean (SD)	48.88 (17.52)	46.29 (17.00)
Testosterone, ng/dl, mean (SD)	409.29 (180.01)	24.82 (22.81)
Estradiol, pg/ml, mean (SD)	24.52 (9.59),	82.91 (383.58),
SHBG, nmol/l, mean (SD)	44.41 (26.13)	78.19 (71.65)
Time of venipuncture		
Morning	1179 (47.93%)	1002 (45.67%)
Afternoon	899 (36.54%)	813 (37.06%)
Evening	382 (15.53%)	379 (17.27%)
Race, n (%)		
Mexican American	330 (13.41%)	336 (15.31%)
Other Hispanic	244 (9.92%)	277 (12.63%)
Non-Hispanic White	955 (38.82%)	813 (37.06%)
Non-Hispanic Black	537 (21.83%)	413 (18.82%)
Other Race	394 (16.02%)	355 (16.18%)
Ratio of family income to poverty, n (%)		
<1.3	712 (28.94%)	679 (30.95%)
1.3-3.5	1039 (42.24%)	909 (41.43%)
>3.5	709 (28.82%)	606 (27.62%)
Education level, n (%)		
Less than high school	595 (24.19%)	439 (20.01%)
High school or GED	556 (22.60%)	435 (19.83%)
Above high school	1309 (53.21%)	1320 (60.16%)
Marital, n (%)		
Married or living with partner	1590 (64.63%)	1235 (56.29%)
Living alone	870 (35.37%)	959 (43.71%)
BMI, n (%)		
<=25	723 (29.39%)	688 (31.36%)
>25, <=30	911 (37.03%)	590 (26.89%)
>30	826 (33.58%)	916 (41.75%)
Comorbidity index, n (%)		
0	1450 (58.94%)	1354 (61.71%)
1	731 (29.72%)	647 (29.49%)
2, 3, 4, 5	279 (11.34%)	193 (8.80%)
Smoking, n (%)		
Never	1167 (47.44%)	1477 (67.32%)
Former	864 (35.12%)	424 (19.33%)
Current	429 (17.44%)	293 (13.35%)
Alcohol intake, n (%)		
none	1788 (72.68%)	1841 (83.91%)
moderate	254 (10.33%)	81 (3.69%)
heavy	418 (16.99%)	272 (12.40%)

SD, standard deviation; GED, general educational development; BMI, body mass index, SHBG, sex hormone-binding globulin.

### PAH metabolite levels

**Table 2** showed the geometric mean and creatine-corrected mean concentration of 10 urinary PAH metabolites. Urinary 1-hydroxynapthalene and 2-hydroxynapthalene metabolites accounted for almost 90% of total urinary PAH metabolites. Besides, urinary 2-hydroxyfluorene and 9-hydroxyfluorene metabolites were higher than the rest PAH metabolites.

**Table 2:** Distribution of urinary PAH metabolites among 4,654 subjects.

Urinary exposure biomarkers	Geometric mean (95%CI)	Creatinine-corrected geometric mean (95%CI)
1-hydroxynapthalene	1880.85 (1800.07-1965.26)	2055.58 (1973.07-2141.53)
2-hydroxynapthalene	4984.40 (4824.23-5149.89)	5455.18 (5314.46-5599.63)
3-hydroxyfluorene	93.68 (90.14-97.37)	102.27 (98.94-105.71)
2-hydroxyfluorene	224.08 (216.56-231.86)	244.62 (237.91-251.52)
3-hydroxyphenanthrene	65.71 (62.33-69.26)	70.90 (68.03-73.89)
1-hydroxyphenanthrene	109.03 (106.17-111.95)	119.03 (116.62-121.48)
2-hydroxyphenanthrene	66.86 (63.80-70.08)	72.08 (69.49-74.78)
1-hydroxypyrene	120.38 (117.22-123.64)	131.51 (128.47-134.63)
9-hydroxyfluorene	270.18 (255.95-285.19)	291.56 (279.00-304.69)
4-hydroxyphenanthrene	21.54 (20.59-22.54)	23.23 (22.32-24.18)

PAH, polycyclic aromatic hydrocarbon; CI, confidence interval.

### Association between urinary PAH metabolites and serum T levels of males

The associations between 10 types of urinary PAH metabolites and serum T levels of males are shown in **Table 3**. In the non-adjusted model, 1-hydroxynapthalene, 2-hydroxynapthalene, 3-hydroxyfluorene, 2-hydroxyfluorene, 3-hydroxyphenanthrene, and 1-hydroxypyrene were associated with increased serum T levels of males (p-value for trends were all <0.05). After adjusting for confounders, 2-hydroxynapthalene and 3-hydroxyfluorene remained positively correlated with serum T level [βs for increasing quartiles: 0, -2.74 (95%CI -21.18 to 15.70), 0.49 (95%CI -18.65 to 19.63), 18.08 (95%CI -3.23 to 39.39), p-value for trend=0.047; and βs for increasing quartiles: 0, 1.43 (95%CI -16.92 to 19.78), -2.71 (95%CI -21.24 to 15.83), 29.13 (95%CI 5.14 to 53.12), p-value for trend=0.006, respectively]. No significant association between PAHs metabolites and SHBG was found (**Supplementary Table 1**). In the age-stratified analysis, 2-hydroxynapthalene was positively associated with serum T levels among males aged 40-60 (p-value for trend=0.007), while the positive association was found between 3-hydroxyfluorene and serum T among males aged 20-40 (p-value for trend=0.002) (**Supplementary Table 2**). The correlation between PAHs scores and serum T levels of males were shown in **Table 4**. In non-adjusted model, serum T levels were positively associated with PAHs score (p-value for trend=0.031). However, after adjusting for confounders, the association did not remain significant (p-value for trend=0.615). In addition, no significant association was found between PAHs scores and serum T levels of males in the three different age groups (**Supplementary Table 4**).

**Table 3:** Relationships between urinary PAH metabolites and sex hormones in the non-adjusted and adjusted model.

			Testosterone (ng/dl)		Estradiol (pg/ml)	
Exposure biomarkers			β (95%CI)	p-value	β (95%CI)	p-value
1-hydroxynapthalene	Non-adjusted	Quartile 1	0		0	
		Quartile 2	-4.00 (-24.15, 16.14)	0.697	11.93 (-41.68, 65.54)	0.663
		Quartile 3	4.42 (-15.73, 24.58)	0.667	0.28 (-54.28, 54.84)	0.992
		Quartile 4	50.77 (30.62, 70.92)	<0.001	21.09 (-33.10, 75.27)	0.446
		p-value for trend		<0.001		0.466
	Adjusted	Quartile 1	0		0	
		Quartile 2	0.60 (-17.89, 19.09)	0.949	31.50 (-22.12, 85.12)	0.250
		Quartile 3	-6.16 (-24.98, 12.65)	0.521	31.50 (-23.96, 86.95)	0.266
		Quartile 4	10.51 (-11.95, 32.97)	0.359	32.59 (-32.53, 97.72)	0.327
		p-value for trend		0.230		0.615
2-hydroxynapthalene	Non-adjusted	Quartile 1	0		0	
		Quartile 2	-6.94 (-27.02, 13.14)	0.498	16.04 (-37.29, 69.37)	0.556
		Quartile 3	-0.38 (-20.45, 19.70)	0.971	33.41 (-19.51, 86.32)	0.216
		Quartile 4	37.98 (17.91, 58.04)	<0.001	77.64 (24.66, 130.61)	0.004
		p-value for trend		<0.001		0.002
	Adjusted	Quartile 1	0		0	
		Quartile 2	-2.74 (-21.18, 15.70)	0.771	-8.01 (-62.11, 46.08)	0.772
		Quartile 3	0.49 (-18.65, 19.63)	0.960	-13.66 (-69.59, 42.28)	0.632
		Quartile 4	18.08 (-3.23, 39.39)	0.096	36.56 (-23.98, 97.11)	0.237
		p-value for trend		0.047		0.141
3-hydroxyfluorene	Non-adjusted	Quartile 1	0		0	
		Quartile 2	11.08 (-8.81, 30.96)	0.275	7.68 (-43.92, 59.28)	0.770
		Quartile 3	16.97 (-2.77, 36.71)	0.092	45.11 (-6.11, 96.33)	0.085
		Quartile 4	85.29 (65.49, 105.09)	<0.001	57.03 (4.74, 109.31)	0.033
		p-value for trend		<0.001		0.045
	Adjusted	Quartile 1	0		0	
		Quartile 2	1.43 (-16.92, 19.78)	0.879	-0.19 (-51.44, 51.06)	0.994
		Quartile 3	-2.71 (-21.24, 15.83)	0.775	33.57 (-17.79, 84.92)	0.200
		Quartile 4	29.13 (5.14, 53.12)	0.017	23.90 (-42.28, 90.07)	0.479
		p-value for trend		0.006		0.521
2-hydroxyfluorene	Non-adjusted	Quartile 1	0		0	
		Quartile 2	-7.06 (-26.99, 12.87)	0.488	14.44 (-36.39, 65.27)	0.578
		Quartile 3	0.99 (-18.94, 20.93)	0.922	46.91 (-4.35, 98.18)	0.073
		Quartile 4	57.86 (37.94, 77.79)	<0.001	72.83 (20.93, 124.74)	0.006
		p-value for trend		<0.001		0.008
	Adjusted	Quartile 1	0		0	
		Quartile 2	-5.78 (-24.07, 12.51)	0.536	3.43 (-47.12, 53.98)	0.894
		Quartile 3	-6.24 (-24.83, 12.36)	0.511	31.47 (-19.93, 82.87)	0.230
		Quartile 4	8.09 (-15.04, 31.23)	0.493	48.75 (-17.40, 114.90)	0.149
		p-value for trend		0.275		0.143
3-hydroxyphenanthrene	Non-adjusted	Quartile 1	0			
		Quartile 2	5.77 (-27.59, 39.13)	0.735		
		Quartile 3	26.17 (-7.19, 59.53)	0.125		
		Quartile 4	45.21 (11.84, 78.57)	0.008		
		p-value for trend		0.004		
	Adjusted	Quartile 1	0			
		Quartile 2	-9.49 (-40.79, 21.81)	0.552		
		Quartile 3	3.32 (-28.75, 35.38)	0.839		
		Quartile 4	8.61 (-26.44, 43.67)	0.630		
		p-value for trend		0.455		
1-hydroxyphenanthrene	Non-adjusted	Quartile 1	0		0	
		Quartile 2	9.29 (-10.86, 29.44)	0.366	-1.46 (-52.09, 49.18)	0.955
		Quartile 3	9.86 (-10.27, 30.00)	0.337	17.92 (-33.45, 69.29)	0.494
		Quartile 4	10.21 (-9.93, 30.34)	0.320	50.82 (-2.231, 103.88)	0.061
		p-value for trend		0.446		0.034
	Adjusted	Quartile 1	0		0	
		Quartile 2	3.82 (-14.57, 22.21)	0.684	1.94 (-48.71, 52.59)	0.940
		Quartile 3	-1.32 (-19.89, 17.24)	0.889	24.68 (-27.23, 76.59)	0.352
		Quartile 4	-10.49 (-29.82, 8.83)	0.287	63.94 (7.73, 120.14)	0.026
		p-value for trend		0.167		0.013
2-hydroxyphenanthrene	Non-adjusted	Quartile 1	0			
		Quartile 2	23.76 (-9.78, 57.31)	0.165		
		Quartile 3	13.81 (-19.69, 47.31)	0.419		
		Quartile 4	16.14 (-17.36, 49.64)	0.345		
		p-value for trend		0.637		
	Adjusted	Quartile 1	0			
		Quartile 2	20.40 (-11.23, 52.04)	0.207		
		Quartile 3	6.28 (-25.56, 38.11)	0.699		
		Quartile 4	7.34 (-26.49, 41.17)	0.671		
		p-value for trend		0.985		
1-hydroxypyrene	Non-adjusted	Quartile 1	0		0	
		Quartile 2	28.37 (8.42, 48.33)	0.005	15.47 (-37.10, 68.05)	0.564
		Quartile 3	38.64 (18.68, 58.60)	<0.001	18.21 (-34.67, 71.09)	0.500
		Quartile 4	70.43 (50.48, 90.38)	<0.001	48.46 (-4.29, 101.21)	0.072
		p-value for trend		<0.001		0.068
	Adjusted	Quartile 1	0		0	
		Quartile 2	18.46 (0.06, 36.87)	0.049	5.40 (-46.88, 57.67)	0.840
		Quartile 3	23.49 (4.80, 42.19)	0.014	-0.44 (-37.54, 52.73)	0.987
		Quartile 4	25.51 (5.28, 45.75)	0.014	19.54 (-41.24, 76.63)	0.502
		p-value for trend		0.052		0.495
9-hydroxyfluorene	Non-adjusted	Quartile 1	0			
		Quartile 2	4.20 (-29.27, 37.66)	0.806		
		Quartile 3	-19.26 (-52.73, 14.21)	0.260		
		Quartile 4	12.83 (-20.64, 46.30)	0.453		
		p-value for trend		0.394		
	Adjusted	Quartile 1	0			
		Quartile 2	-2.84 (-34.36, 28.68)	0.860		
		Quartile 3	-18.40 (-50.43, 13.63)	0.261		
		Quartile 4	3.26 (-31.93, 38.44)	0.856		
		p-value for trend		0.718		
4-hydroxyphenanthrene	Non-adjusted	Quartile 1	0			
		Quartile 2	-21.42 (-54.86, 12.01)	0.210		
		Quartile 3	-21.16 (-54.60, 12.28)	0.215		
		Quartile 4	-12.03 (-45.43, 21.36)	0.480		
		p-value for trend		0.788		
	Adjusted	Quartile 1	0			
		Quartile 2	-12.16 (-43.21, 18.89)	0.443		
		Quartile 3	-15.84 (-47.05, 15.37)	0.320		
		Quartile 4	-15.76 (-49.27, 17.75)	0.357		
		p-value for trend		0.457		

Adjusted model: adjusted for age, race, BMI, time of venipuncture, ratio of family income to poverty, education level, marital, comorbidity index, smoking, and alcohol intake.

CI, confidence interval.

**Table 4:** Relationships between PAHs scores and sex hormones in the non-adjusted and adjusted model.

	Testosterone, ng/dl				Estradiol, pg/ml
	Non-adjusted		Adjusted		Non-adjusted		Adjusted
PAH scores group	β (95%CI)	p-value	β (95%CI)	p-value	β (95%CI)	p-value	β (95%CI)	p-value
Group 1	0		0		0		0	
Group 2	-4.97 (-25.03, 15.09)	0.627	-0.17 (-19.99, 19.65)	0.987	18.69 (-31.56, 68.94)	0.466	23.00 (-32.88, 78.88)	0.420
Group 3	-16.28 (-36.33, 3.78)	0.112	-11.69 (-33.21, 9.84)	0.287	21.15 (-29.46, 71.77)	0.413	9.03 (-55.44, 73.50)	0.784
Group 4	26.22 (6.16, 46.28)	0.010	-2.99 (-28.14, 22.16)	0.816	82.72 (28.46, 136.97)	0.003	64.85 (-19.90, 149.60)	0.134
p-value for trend		0.031		0.615		0.005		0.241

Adjusted model: adjusted for age, race, BMI, time of venipuncture, ratio of family income to poverty, education level, marital, comorbidity index, smoking, and alcohol intake.

CI, confidence interval.

### Association between urinary PAH metabolites and serum E2 levels of females

6 out of 10 urinary PAH metabolites showed liner relationships with serum E2 levels of females, including 1-hydroxynapthalene, 2-hydroxynapthalene, 3-hydroxyfluorene, 2-hydroxyfluorene, 1-hydroxyphenanthrene, and 1-hydroxypyrene (**Table 3**). In the non-adjusted model, 2-hydroxynapthalene, 2-hydroxyfluorene, 3-hydroxyfluorene, and 1-hydroxyphenanthrene were positively associated with serum E2 levels (p=0.002, p=0.045, p=0.008, and p=0.034, respectively). After adjusting for confounders, 1-hydroxyphenanthrene remained positively associated with serum E2 levels [βs for increasing quartiles: 0, 1.94 (95%CI -48.71 to 52.59), 24.68 (95%CI -27.23 to 76.59), 63.94 (95%CI 7.73 to 120.14), p-value for trend=0.013]. In the age-stratified analysis, 1-hydroxyphenanthrene was positively associated with serum E2 levels among females aged 20-40 (p-value for trend=0.002) (**Supplementary Table 3**). The correlation between PAHs scores and serum E2 levels were shown in **Table 4**. In non-adjusted model, serum E2 levels were positively associated with PAHs scores (p-value for trend=0.005). Similarly, after adjusting for confounders, the association did not remain significant (p-value for trend=0.241), and no significant association was found between PAHs scores and serum E2 levels of females in three different age groups (**Supplementary Table 4**).

## Discussion

We examined the association between urinary PAH metabolites and serum T levels of males and serum E2 levels of females separately. The results of this study showed urinary 2-hydroxynapthalene and 3-hydroxyfluorene were positively associated with serum T levels of males, and urinary 1-hydroxyphenanthrene was positively associated with serum E2 levels of females. 2-hydroxynapthalene, 3-hydroxyfluorene, and 1-hydroxyphenanthrene were major metabolites of naphthalene, fluorene, and phenanthrene respectively. This indicated exposure to naphthalene and fluorene might result in an increased level of T of males, and phenanthrene might cause the increasing of E2 level of females.

In the stratified analysis, urinary 2-hydroxynapthalene and 3-hydroxyfluorene were positively associated with serum T levels of males aged 40-60 and 20-40 respectively, and urinary 1-hydroxyphenanthrene was positively associated with serum E2 levels of females aged 20-40. This indicated that young and middle-aged people were more sensitive to the exposure of PAHs than the elderly. PAHs scores was not significantly associated with sex hormones in the three different age groups, which was consistent with the results of total population.

Previous studies focusing on the relationship between PAH exposure and serum sexual hormone levels did not reach a consistent result. In addition, as PAHs consist of hundreds of compounds, the types of PAHs or their metabolites included in different studies were not the same, which made it more difficult to collate and compare the results of different studies. A cross-sectional study which included 371 men in an infertility clinic in Wuhan, China observed 2-hydroxynaphthalene was associated with decreased serum free T (p-value for trend=0.01) (23). However, in another study including 642 males which analyzed the associations between PAH metabolites and several different reproductive hormones, no significant correlation was found between 2-hydroxynapthalene metabolites and serum T levels (24). Interestingly, we observed a positive association between urinary 2-hydroxynapthalene and serum T levels of males in our study. Similarly, results of present studies investigating the association between PAH exposure and serum E2 levels showed a divergence. One study including 120 pregnant Chinese women detected the exposure to both low-molecular-weight and high-molecular weight PAHs could negatively affect the E2 level in the umbilical cord serum (25). There were studies showed a positive association between PAHs exposure and serum E2 level. In one study including 122 non-smoker males, there was a positive association between hydroxypyrene and serum E2 levels (26). Benefiting from NHANES database, we included more kinds of PAH metabolites, and the sample size of our study was larger compared with previous studies, which made our results more systematic and reliable.

The positive association between PAHs exposure and serum T and E2 levels may be explained by several mechanisms. One study explored the association between GnRH expression and short-term benzo(a)pyrene (one type of PAH) exposure, which showed benzo(a)pyrene was positively associated with GnRH by increased the mRNA expression of GnRH2 and GnRH3 (27). This indicated some kinds of PAHs could promote the secretion of GnRH, and furtherly promote the secretion of LT and T. Another study explored the alteration of sex hormone by PAH in rats and a significant increase of LH was observed after exposing to PAHs. In this study, serum T levels were decreased during PAH exposure, while T levels were gradually increased within 48 hours after the exposure. At 72h, the T levels were higher than control group (28). This could be explained by that remaining vital Leydig cells conducted a compensatory synthesis and release of T, and this likely be another reason why PAH metabolites were positively associated with serum T in our study. The positive association between PAHs exposure and serum E2 levels can also be explained by the hypothalamic-pituitary-gonadal axis. Previous studies reported exposure to PAHs was related to increased levels of FSH and LH (24), which could furtherly lead to an increase of serum E2 levels. The monohydroxy derivatives of some PAHs have similar structures with 17β-estradiol, and some of the PAH metabolites can interact with estrogen receptors, demonstrating anti-estrogenic activity (29). This can lead to an increased secretion of E2 to maintain the normal functions of estrogenic hormones.

Our study still has some limitations. First, given the characteristics of cross-sectional design, we cannot make any conclusion about causal link between PAH exposure and serum T and E2 levels. Second, as an observational study, although a number of covariates have been adjusted, unmeasured confounding factors still cannot be excluded. Third, as the half-life of PAHs are short, the urinary PAH metabolites show a within-subject variability over time, which may result in a slight difference from the actual exposure levels (30).

In conclusion, association between PAHs exposure and sex hormones varied by specific PAHs. This study showed urinary 2-hydroxynapthalene and 3-hydroxyfluorene were associated with increased T levels of males, and urinary 1-hydroxyphenanthrene was associated with increased E2 levels of females. The observed association indicated disrupting effects of PAH exposure on reproductive health.

## Data availability statement

Publicly available datasets were analyzed in this study. This data can be found here: https://wwwn.cdc.gov/nchs/nhanes/default.aspx.

## Ethics statement

The studies involving human participants were reviewed and approved by the National Center for Health Statistics Research Ethics Review Board. The patients/participants provided their written informed consent to participate in this study.

## Author contributions

QY: Data analysis, Manuscript writing. KJ: Data analysis, Manuscript writing. XZ: Data management, Data analysis. ZQ: Data collection and management. JL: Data collection and management. DJ: Data curation, Validation. ZZ Data collection. CZ: Data collection. LY: Manuscript editing. YZ: Manuscript editing. SQ: Project development, Manuscript editing. QW: Project development, Manuscript editing. All authors contributed to the article and approved the submitted version.

## Funding

This work was supported by the National key research and development program of China (Grant No. 2017YFC0908003), National Natural Science Foundation of China (Grant No.81902578, 81974098), China Postdoctoral Science Foundation (2017M612971), Post-doctoral Science Research Foundation of Sichuan University (2020SCU12041), Post-Doctor Research Project, West China Hospital, Sichuan University (2018HXBH085), National Clinical Research Center for Geriatrics, West China Hospital, Sichuan University (Z2018C01).

## Acknowledgments

The authors gratefully thank Dr. Changzhong Chen, Chi Chen, and Xin-Lin Chen (EmpowerStats X&Y Solutions, Inc., Boston, MA) for providing statistical methodology consultation.

## Conflict of interest

The authors declare that the research was conducted in the absence of any commercial or financial relationships that could be construed as a potential conflict of interest.

## Publisher’s note

All claims expressed in this article are solely those of the authors and do not necessarily represent those of their affiliated organizations, or those of the publisher, the editors and the reviewers. Any product that may be evaluated in this article, or claim that may be made by its manufacturer, is not guaranteed or endorsed by the publisher.
