# Supplementary material for: Urinary polycyclic aromatic hydrocarbon metabolites are positively related to serum testosterone levels of males and serum estradiol levels of females among U.S. adults
Source: Front Endocrinol (Lausanne). 2022 Dec 7;13:1037098. doi: 10.3389/fendo.2022.1037098 (PMC9768547; doi:10.3389/fendo.2022.1037098)
Supplement: Supplementary file 1 [file Table_1.docx]

**Supplementary Table 1. Relationships between PAH metabolites and sex hormone binding globulin of males**

|  | | | SHBG (nmol/l) | |
| --- | --- | --- | --- | --- |
| Exposure biomarkers | | | β (95%CI) | p-value |
| 1-hydroxynapthalene | Quartile 1 | 0 | |  |
|  | Quartile 2 | -0.12 (-3.25, 3.01) | | 0.940 |
|  | Quartile 3 | -0.60 (-3.88, 2.68) | | 0.720 |
|  | Quartile 4 | 1.57 (-2.35, 5.48) | | 0.433 |
|  | p-value for trend | | | 0.305 |
| 2-hydroxynapthalene | Quartile 1 | 0 | |  |
|  | Quartile 2 | 0.00 (-3.24, 3.25) | | 0.998 |
|  | Quartile 3 | 0.79 (-2.53, 4.11) | | 0.639 |
|  | Quartile 4 | 3.07 (-0.56, 6.69) | | 0.098 |
|  | p-value for trend | | | 0.058 |
| 3-hydroxyfluorene | Quartile 1 | 0 | |  |
|  | Quartile 2 | 0.61 (-2.48, 3.71) | | 0.699 |
|  | Quartile 3 | 0.91 (-2.30, 4.13) | | 0.579 |
|  | Quartile 4 | 3.92 (-0.17, 8.00) | | 0.061 |
|  | p-value for trend | | | 0.057 |
| 2-hydroxyfluorene | Quartile 1 | 0 | |  |
|  | Quartile 2 | 0.79 (-2.23, 3.81) | | 0.607 |
|  | Quartile 3 | 0.44 (-2.77, 3.65) | | 0.789 |
|  | Quartile 4 | 2.82 (-1.18, 6.83) | | 0.167 |
|  | p-value for trend | | | 0.173 |
| 3-hydroxyphenanthrene | Quartile 1 |  | |  |
|  | Quartile 2 |  | |  |
|  | Quartile 3 |  | |  |
|  | Quartile 4 |  | |  |
|  | p-value for trend | | | NA |
| 1-hydroxyphenanthrene | Quartile 1 | 0 | |  |
|  | Quartile 2 | -0.65 (-3.69, 2.39) | | 0.675 |
|  | Quartile 3 | -1.16 (-4.29, 1.97) | | 0.469 |
|  | Quartile 4 | -0.21 (-3.50, 3.08) | | 0.901 |
|  | p-value for trend | | | 0.994 |
| 2-hydroxyphenanthrene | Quartile 1 |  | |  |
|  | Quartile 2 |  | |  |
|  | Quartile 3 |  | |  |
|  | Quartile 4 |  | |  |
|  | p-value for trend | | | NA |
| 1-hydroxypyrene | Quartile 1 | 0 | |  |
|  | Quartile 2 | 2.19 (-1.02, 5.40) | | 0.181 |
|  | Quartile 3 | 1.10 (-2.13, 4.33) | | 0.504 |
|  | Quartile 4 | 3.51 (0.01, 7.01) | | 0.050 |
|  | p-value for trend | | | 0.085 |
| 9-hydroxyfluorene | Quartile 1 |  | |  |
|  | Quartile 2 |  | |  |
|  | Quartile 3 |  | |  |
|  | Quartile 4 |  | |  |
|  | p-value for trend | | | NA |
| 4-hydroxyphenanthrene | Quartile 1 |  | |  |
|  | Quartile 2 |  | |  |
|  | Quartile 3 |  | |  |
|  | Quartile 4 |  | |  |
|  | p-value for trend | | | NA |

The model was adjusted for age, race, BMI, time of venipuncture, ratio of family income to poverty, education level, marital, comorbidity index, smoking, and alcohol intake.

Abbreviations

SHBG=sex hormone binding globulin; CI=confidence interval

Supplementary Table 2. Relationship between PAH metabolites and testosterone levels of males in different age groups

|  | | <40 (n=876) | | >=40, <60 (n=799) |  | >60 (n=785) |  |
| --- | --- | --- | --- | --- | --- | --- | --- |
| Exposure biomarkers | | β (95%CI) | p-value | β (95%CI) | p-value | β (95%CI) | p-value |
| 1-hydroxynapthalene | Quartile 1 | 0 |  | 0 |  | 0 |  |
|  | Quartile 2 | -2.61 (-32.78, 27.56) | 0.865 | -4.52 (-37.23, 28.19) | 0.787 | 1.77 (-32.58, 36.11) | 0.920 |
|  | Quartile 3 | -1.00 (-31.66, 29.67) | 0.949 | -10.13 (-43.15, 22.89) | 0.548 | -16.27 (-51.85, 19.32) | 0.371 |
|  | Quartile 4 | 12.27 (-25.42, 49.97) | 0.524 | 18.66 (-21.29, 58.60) | 0.360 | -14.52 (-55.31, 26.28) | 0.486 |
|  | p-value for trend | | 0.433 |  | 0.183 |  | 0.492 |
| 2-hydroxynapthalene | Quartile 1 | 0 |  | 0 |  | 0 |  |
|  | Quartile 2 | 14.33 (-16.67, 45.32) | 0.365 | -13.32 (-46.76, 20.12) | 0.435 | -9.72 (-41.73, 22.28) | 0.552 |
|  | Quartile 3 | -1.31 (-33.57, 30.96) | 0.937 | 5.95 (-28.32, 40.23) | 0.734 | 0.80 (-32.59, 34.20) | 0.962 |
|  | Quartile 4 | 14.22 (-21.41, 49.86) | 0.434 | 39.23 (1.67, 76.80) | 0.041 | -9.61 (-49.40, 30.18) | 0.636 |
|  | p-value for trend | | 0.569 |  | 0.007 |  | 0.741 |
| 3-hydroxyfluorene | Quartile 1 | 0 |  | 0 |  | 0 |  |
|  | Quartile 2 | 13.24 (-17.80, 44.28) | 0.403 | 0.61 (-32.59, 33.81) | 0.971 | -9.46 (-41.45, 22.53) | 0.562 |
|  | Quartile 3 | 18.45 (-13.44, 50.33) | 0.257 | -1.48 (-33.97, 31.00) | 0.929 | -21.89 (-54.55, 10.77) | 0.189 |
|  | Quartile 4 | 62.49 (23.24, 101.74) | 0.002 | 18.81 (-22.57, 60.19) | 0.373 | -3.58 (-51.04, 43.87) | 0.882 |
|  | p-value for trend | | 0.002 |  | 0.295 |  | 0.898 |
| 2-hydroxyfluorene | Quartile 1 | 0 |  | 0 |  | 0 |  |
|  | Quartile 2 | -8.52 (-39.13, 22.09) | 0.586 | -6.20 (-39.56, 27.16) | 0.716 | 3.54 (-28.55, 35.64) | 0.829 |
|  | Quartile 3 | 8.18 (-23.62, 39.97) | 0.614 | -19,87 (-53.24, 13.49) | 0.243 | -4.87 (-37.38, 27.64) | 0.769 |
|  | Quartile 4 | 26.18 (-12.70, 65.06) | 0.187 | 8.47 (-30.92, 47.87) | 0.673 | -16.27 (-61.43, 28.88) | 0.480 |
|  | p-value for trend | | 0.099 |  | 0.360 |  | 0.420 |
| 3-hydroxyphenanthrene | Quartile 1 | 0 |  | 0 |  | 0 |  |
|  | Quartile 2 | 14.43 (-35.45, 64.31) | 0.571 | -13.02 (-70.05, 44.01) | 0.655 | -37.03 (-99.37, 25.31) | 0.246 |
|  | Quartile 3 | 15.99 (-32.32, 64.29) | 0.517 | 22.19 (-34.45, 78.82) | 0.443 | -23.14 (-91.05, 44.77) | 0.505 |
|  | Quartile 4 | 44.21 (-13.66, 102.08) | 0.136 | 11.81 (-49.15, 72.78) | 0.704 | -27.30 (-96.59, 41.99) | 0.441 |
|  | p-value for trend | | 0.145 |  | 0.666 |  | 0.712 |
| 1-hydroxyphenanthrene | Quartile 1 | 0 |  | 0 |  | 0 |  |
|  | Quartile 2 | -4.63 (-33.93, 24.67) | 0.757 | -4.71 (-38.12, 28.69) | 0.782 | 28.68 (-4.89, 62.25) | 0.094 |
|  | Quartile 3 | 20.88 (-9.30, 51.07) | 0.175 | -20.43 (-53.36, 12.49) | 0.224 | -2.28 (-36.40, 31.84) | 0.896 |
|  | Quartile 4 | -0.26 (-33.39, 32.87) | 0.988 | -14.66 (-47.72, 18.40) | 0.385 | -25.12 (-60.43, 10.18) | 0.163 |
|  | p-value for trend | | 0.867 |  | 0.415 |  | 0.020 |
| 2-hydroxyphenanthrene | Quartile 1 | 0 |  | 0 |  | 0 |  |
|  | Quartile 2 | 7.97 (-40.30, 56.25) | 0.746 | 70.68 (13.52, 127.84) | 0.016 | 5.79 (-58.50, 70.08) | 0.860 |
|  | Quartile 3 | 17.00 (-33.63, 67.64) | 0.511 | 14.75 (-42.38, 71.88) | 0.613 | 12.61 (-52.30, 77.52) | 0.704 |
|  | Quartile 4 | 64.15 (7.14, 121.15) | 0.028 | 10.31 (-48.78, 69.40) | 0.733 | -38.11 (-102.39, 26.17) | 0.246 |
|  | p-value for trend | | 0.018 |  | 0.518 |  | 0.178 |
| 1-hydroxypyrene | Quartile 1 | 0 |  | 0 |  | 0 |  |
|  | Quartile 2 | 35.97 (4.64, 67.30) | 0.025 | 15.79 (-18.12, 49.70) | 0.362 | 6.20 (-25.21, 37.60) | 0.699 |
|  | Quartile 3 | 52.63 (20.23, 85.03) | 0.002 | 7.11 (-26.11, 40.34) | 0.675 | 8.90 (-23.40, 41.20) | 0.589 |
|  | Quartile 4 | 52.17 (17.38, 86.96) | 0.003 | 23.84 (-10.72, 58.39) | 0.177 | -2.80 (-40.67, 35.07) | 0.885 |
|  | p-value for trend | | 0.029 |  | 0.227 |  | 0.823 |
| 9-hydroxyfluorene | Quartile 1 | 0 |  | 0 |  | 0 |  |
|  | Quartile 2 | -1.99 (-48.25, 44.26) | 0.933 | -20.95 (-78.86, 36.95) | 0.479 | 14.29 (-52.24, 80.83) | 0.674 |
|  | Quartile 3 | -10.27 (-59.92, 39.38) | 0.685 | -15.95 (-72.61, 40,71) | 0.582 | -30.61 (-97.33, 36.12) | 0.370 |
|  | Quartile 4 | 17.35 (-41.76, 76.46) | 0.566 | 15.77 (-43.69, 75.24) | 0.604 | -24.92 (-98.12, 48.28) | 0.505 |
|  | p-value for trend | | 0.511 |  | 0.342 |  | 0.412 |
| 4-hydroxyphenanthrene | Quartile 1 | 0 |  | 0 |  | 0 |  |
|  | Quartile 2 | -4.92 (-54.80, 44.96) | 0.847 | -13.16 (-69.64, 43.32) | 0.648 | -8.25 (-69.55, 53.06) | 0.792 |
|  | Quartile 3 | 3.40 (-45.65, 52.45) | 0.892 | -2.82 (-61.89, 56.25) | 0.926 | -35.78 (-98.56, 27.00) | 0.265 |
|  | Quartile 4 | 17.25 (-40.57, 75.07) | 0.559 | -31.06 (-88.05, 25.93) | 0.287 | -29.33 (-98.41, 39.74) | 0.406 |
|  | p-value for trend | | 0.455 |  | 0.277 |  | 0.385 |

The model was adjusted for race, BMI, time of venipuncture, ratio of family income to poverty, education level, marital, comorbidity index, smoking, and alcohol intake.

Abbreviations

CI=confidence interval

Supplementary Table 3. Relationship between PAH metabolites and estradiol levels of females in different age groups

|  | | <40 (n=881) | | >=40, <60 (n=757) |  | >60 (n=556) |  |
| --- | --- | --- | --- | --- | --- | --- | --- |
| Exposure biomarkers | | β (95%CI) | p-value | β (95%CI) | p-value | β (95%CI) | p-value |
| 1-hydroxynapthalene | Quartile 1 | 0 |  | 0 |  | 0 |  |
|  | Quartile 2 | 58.23 (-74.65, 191.11) | 0.391 | 2.15 (-62.42, 66.71) | 0.948 | 0.04 (-2.25, 2.33) | 0.974 |
|  | Quartile 3 | 8.36 (-134.52, 151.25) | 0.909 | 29.58 (-36.04, 95.20) | 0.377 | -0.26 (-2.58, 2.05) | 0.823 |
|  | Quartile 4 | 12.13 (-162.17, 186.42) | 0.892 | 58.96 (-18.70, 136.62) | 0.137 | -1.68 (-4.30, 0.93) | 0.207 |
|  | p-value for trend | | 0.913 |  | 0.122 |  | 0.116 |
| 2-hydroxynapthalene | Quartile 1 | 0 |  | 0 |  | 0 |  |
|  | Quartile 2 | 4.33 (-146.33, 154.99) | 0.955 | 5.71 (-62.45, 73.86) | 0.870 | 0.66 (-1.26, 2.57) | 0.500 |
|  | Quartile 3 | 8.38 (-141.24, 157.99) | 0.913 | -6.10 (-73.70, 61.51) | 0.860 | -1.42 (-3.65, 0.80) | 0.210 |
|  | Quartile 4 | 74.60 (-85.07, 234.27) | 0.360 | 47.93 (-28.19, 124.05) | 0.218 | 0.07 (-2.30, 2.43) | 0.956 |
|  | p-value for trend | | 0.279 |  | 0.164 |  | 0.836 |
| 3-hydroxyfluorene | Quartile 1 | 0 |  | 0 |  | 0 |  |
|  | Quartile 2 | -18.21 (-163.86, 127.43) | 0.806 | 4.91 (-55.57, 65.38) | 0.874 | -0.82 (-2.73, 1.09) | 0.398 |
|  | Quartile 3 | 73.63 (-69.51, 216.78) | 0.314 | 22.85 (-37.83, 83.53) | 0.461 | -1.19 (-3.15, 0.77) | 0.234 |
|  | Quartile 4 | 29.82 (-144.97, 204.60) | 0.738 | 53.89 (-25.44, 133.22) | 0.184 | -0.83 (-3.52, 1.85) | 0.542 |
|  | p-value for trend | | 0.762 |  | 0.182 |  | 0.702 |
| 2-hydroxyfluorene | Quartile 1 | 0 |  | 0 |  | 0 |  |
|  | Quartile 2 | 18.45 (-121.47, 158.37) | 0.796 | -5.80 (-67.59, 55.99) | 0.854 | -0.00 (-1.88, 1.88) | 0.997 |
|  | Quartile 3 | 47.84 (-92.99, 188.67) | 0.506 | 50.30 (-10.16, 110.76) | 0.104 | -0.45 (-2.46, 1.56) | 0.661 |
|  | Quartile 4 | 163.24 (-12.49, 338.96) | 0.069 | -29.10 (-108.12, 49.91) | 0.471 | -0.50 (-3.14, 2.13) | 0.708 |
|  | p-value for trend | | 0.056 |  | 0.407 |  | 0.693 |
| 1-hydroxyphenanthrene | Quartile 1 | 0 |  | 0 |  | 0 |  |
|  | Quartile 2 | 17.74 (-112.46, 147.94) | 0.789 | -31.84 (-93.16, 29.49) | 0.309 | -1.36 (-3.37, 0.65) | 0.185 |
|  | Quartile 3 | 67.33 (71.09, 205.76) | 0.341 | -26.81 (-88.80, 35.18) | 0.397 | -1.13 (-3.19, 0.93) | 0.283 |
|  | Quartile 4 | 221.58 (73.95, 369.21) | 0.003 | -50.32 (-120.51, 19.88) | 0.161 | -1.43 (-3.58, 0.71) | 0.190 |
|  | p-value for trend | | 0.002 |  | 0.221 |  | 0.297 |
| 1-hydroxypyrene | Quartile 1 | 0 |  | 0 |  | 0 |  |
|  | Quartile 2 | 40.82 (-110.77, 192.42) | 0.598 | -9.77 (-71.03, 51.50) | 0.755 | -1.71 (-3.62, 0.20) | 0.080 |
|  | Quartile 3 | 53.35 (-97.51, 204.22) | 0.489 | -31.66 (-94.60, 31.29) | 0.325 | -0.95 (-2.96, 1.06) | 0.354 |
|  | Quartile 4 | 109.47 (-45.84, 264.77) | 0.168 | -48.07 (-116.45, 20.31) | 0.169 | -1.09 (-3.36, 1.18) | 0.347 |
|  | p-value for trend | | 0.162 |  | 0.154 |  | 0.545 |

The model was adjusted for race, BMI, time of venipuncture, ratio of family income to poverty, education level, marital, comorbidity index, smoking, and alcohol intake.

Abbreviations

CI=confidence interval

Supplementary Table 4. Relationships between PAHs scores and sex hormones in different age groups

| PAH scores group | Testosterone, ng/dl | | | |  |  | Estradiol, pg/ml | | | |  |  |
| --- | --- | --- | --- | --- | --- | --- | --- | --- | --- | --- | --- | --- |
|  | <40 (n=876) | | >=40, <60 (n=799) | | >60 (n=785) | | <40 (n=881) | | >=40, <60 (n=757) | | >60 (n=556) | |
|  | β (95%CI) | p-value | β (95%CI) | p-value | β (95%CI) | p-value | β (95%CI) | p-value | β (95%CI) | p-value | β (95%CI) | p-value |
| Group 1 | 0 |  | 0 |  | 0 |  | 0 |  | 0 |  | 0 |  |
| Group 2 | -16.76 (-50.89, 17.37) | 0.336 | 15.44 (-21,20, 52.09) | 0.409 | 5.05 (-28.52, 38.64) | 0.768 | 77.06 (-80.75, 234.87) | 0.339 | 30.07 (-36.79, 96.93) | 0.378 | -0.60 (-2.66, 1.46) | 0.570 |
| Group 3 | -16.76 (-54.10, 20.59) | 0.379 | 3.88 (-34.04, 41.81) | 0.841 | -24.37 (-62.45, 13.71) | 0.210 | 47.17 (-133.69, 228.03) | 0.609 | -9.31 (-86.94, 68.33) | 0.814 | -0.68 (-3.13, 1.78) | 0.589 |
| Group 4 | -7.37 (-49.76, 35.03) | 0.734 | 10.66 (-33.37, 54.69) | 0.635 | -18.77 (-65.23, 27.70) | 0.429 | 168.49 (-59.59, 396.58) | 0.148 | 45.06 (-54.66, 144.79) | 0.376 | -2.46 (-6.01, 1.08) | 0.174 |
| p-value for trend | | 0.839 |  | 0.826 |  | 0.234 |  | 0.212 |  | 0.648 |  | 0.254 |

The model was adjusted for race, BMI, time of venipuncture, ratio of family income to poverty, education level, marital, comorbidity index, smoking, and alcohol intake.

Abbreviations

CI=confidence interval
